# Supplementary material for: Targeting Pyruvate Kinase M2 and Lactate Dehydrogenase A Is an Effective Combination Strategy for the Treatment of Pancreatic Cancer
Source: Cancers (Basel). 2019 Sep 16;11(9):1372. doi: 10.3390/cancers11091372 (PMC6770573; doi:10.3390/cancers11091372)
Supplement: Supplementary file 1 [file cancers-11-01372-s001.docx]

Supplementary Materials: Targeting Pyruvate Kinase M2 and Lactate Dehydrogenase A Is an Effective Combination Strategy for the Treatment of Pancreatic Cancer

Goran Hamid Mohammad, Vessela Vassileva, Pilar Acedo, Steven W. M. Olde Damink, Massimo Malago, Dipok Kumar Dhar and Stephen P. Pereira


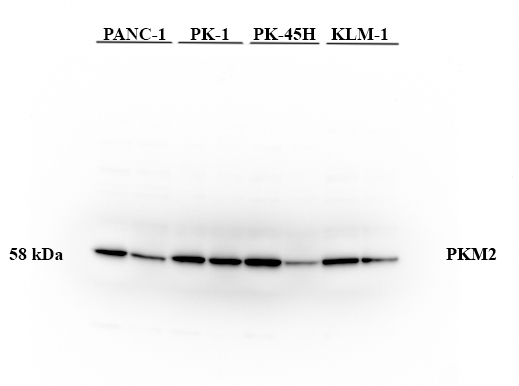


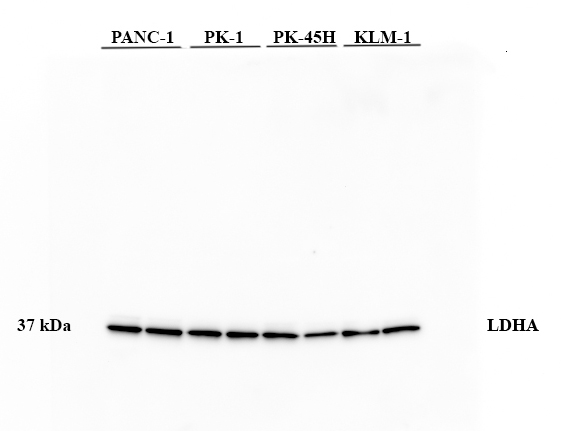


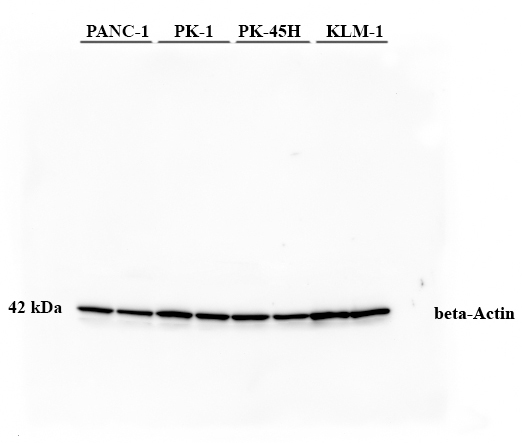


**Figure S1.** Whole plot showing the expression of PKM2 and LDHA in different pancreatic cancer cell lines compared to the loading control (beta-actin). The pictures show the expression of these two glycolytic enzymes in two different stages of cell culture, proliferation (from the left first band, around 50% confluent) and confluent (from the left second band, around 90% confluent) stage.


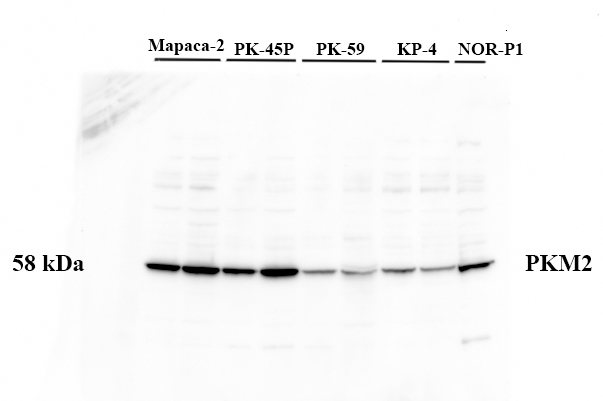


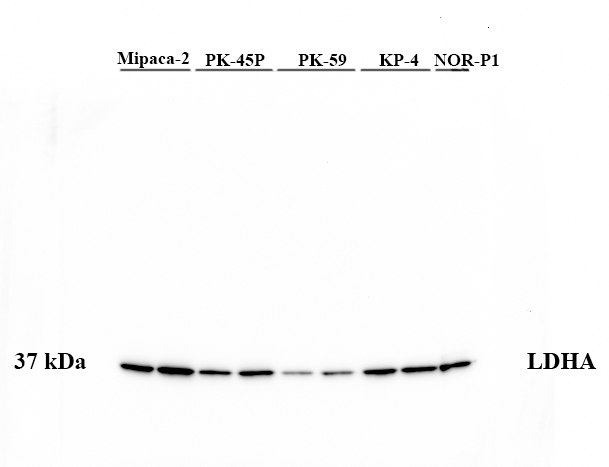


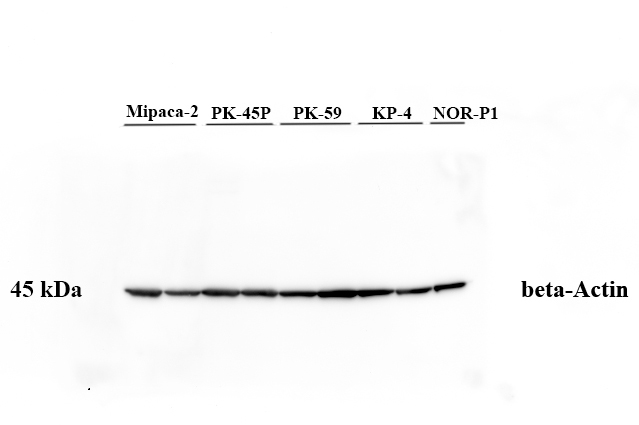


**Figure S2.** Whole plot showing the expression of PKM2 and LDHA in different pancreatic cancer cell lines compared to the loading control (beta-actin). The pictures show the expression of these two glycolytic enzymes in two different stages of cell culture, proliferation (from the left first band, around 50% confluent) and confluent (from the left second band, around 90% confluent) stage.


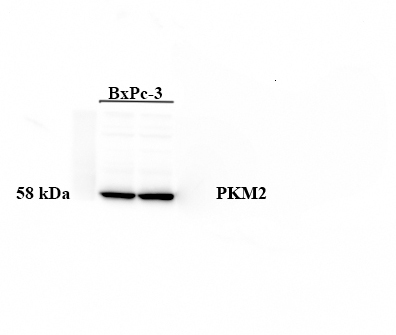


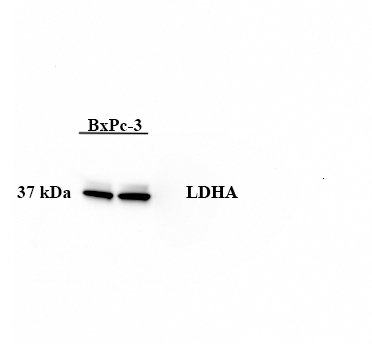


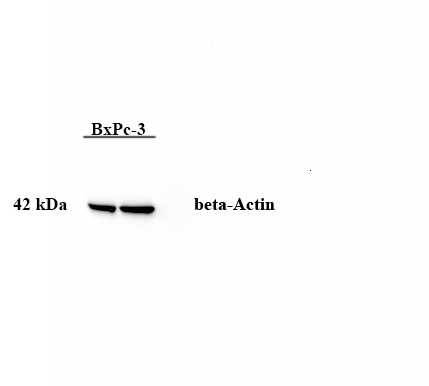


**Figure S3.** Whole plot showing the expression of PKM2 and LDHA compared to the loading control (beta-actin) in the pancreatic cancer cell line BxPc-3. The pictures show the expression of these two glycolytic enzymes in two different stages of cell culture, proliferation (from the left first band, around 50% confluent) and confluent (from the left second band, around 90% confluent) stage.

**Table S1.** Densitometry readings and intensity ratio of each band included in the western blot assays (av.= average).

| 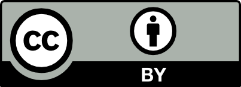 | © 2019 by the authors. Licensee MDPI, Basel, Switzerland. This article is an open access article distributed under the terms and conditions of the Creative Commons Attribution (CC BY) license (http://creativecommons.org/licenses/by/4.0/). |
| --- | --- |
